# Oncological patients' reactions to COVID‐19 pandemic: A single institution prospective study

**DOI:** 10.1002/cnr2.1571

**Published:** 2021-10-12

**Authors:** Concetta Elisa Onesti, Hélène Schroeder, Andrée Rorive, Brieuc Sautois, Marie Lecocq, Marie Goffin, Elodie Gonne, Astrid Collinge, Laurence Nicolaers, Odile Wéra, Amandine Catot, Catherine Loly, Astrid Paulus, Anne Sibille, Laurence Lousberg, Florence Troisfontaine, Joëlle Collignon, Christine Gennigens, Pierre Frères, Marc Polus, Bernard Duysinx, Frédérique Vaillant, Nathalie Marchal, Aurélie Poncin, Guy Jerusalem

**Affiliations:** ^1^ Department of Medical Oncology University Hospital of Liège, CHU Sart Tilman Liège Belgium; ^2^ Laboratory of Human Genetics GIGA Research Center Liège Belgium; ^3^ Department of Gastroenterology University Hospital of Liège, CHU Sart Tilman Liège Belgium; ^4^ Department of Pneumology University Hospital of Liège Liège Belgium; ^5^ University of Liège Liège Belgium

**Keywords:** COVID‐19 and cancer, patients' awareness, patients' feelings, SARS‐CoV2

## Abstract

**Background:**

The spread of the COVID‐19 pandemic has led to a rapid reorganization in all human and hospital activities, with impact on cancer patients.

**Aim:**

An analysis of cancer patients fears, and awareness of COVID‐19 has been done in this study.

**Methods and results:**

We analyzed cancer patients' reactions to the pandemic and their perception of oncological care reorganization, through a 12‐item survey, proposed at the peak of pandemic and 3 months later. Overall, 237 patients were included in the study. During the peak of pandemic 34.6% of patients were more worried about COVID‐19 than cancer versus 26.4% in the post‐acute phase (*p* = .013). Although 49.8% of patients in the acute phase and 42.3% in the post‐acute phase considered their risk of death if infected ≥50%, and more than 70% of patients thought to be at higher risk of complications, the majority of them did not consider the possibility to stop or delay their treatment. Patients were more interested in following news about COVID‐19 than cancer and they complied with all preventive measures in more than 90% of the cases.

**Conclusions:**

Although cancer patients worried about COVID‐19 and evaluated the risk of complication or death due to COVID‐19 as extremely high, they were still asking for the best oncological treatment.

## 1 INTRODUCTION

The spreading of COVID‐19 pandemic, caused by the infection by the coronavirus SARS‐CoV‐2, in the late 2019 led to a health emergency, with a consequent rapid reorganization of all hospital and extra‐hospital activities.^1^ An event of such a magnitude had an important psychological impact on the general population, comparable to a post‐traumatic stress disorder.^2^ This is linked to a concomitance of factors, such as lockdown, interpersonal isolation, fear of contagion, and the economic crisis resulting from the measures taken to contain the virus diffusion, with a greater impact on people with previous psychiatric disorders.^2^

The frailty of cancer patients, the consequent higher risk of complications and mortality, along with the need for treatment and repeated access to the hospital, are the main critical factors in oncological care.^3^, ^4^, ^5^, ^6^, ^7^, ^8^ In fact, the unavailability of resources due to the overload of infected patients and the need to reduce the infectious risk led to a rapid reassessment of oncological care.^9^, ^10^ It has been shown that the disruption of medical activity in oncology, in addition to social isolation, fear of contagion, and economic constraints, has an impact on the psychophysical well‐being of cancer patients, with namely symptoms of depression, anxiety, and sleep disturbances.^11^, ^12^, ^13^, ^14^, ^15^ More than 85% of cancer patients said they worried of cancer progression because of COVID‐19 pandemic.^11^ Trust and proper communication by the medical team have a positive effect on worries related to the delay or interruption of treatment, but more than 80% of patients stated that they do not think the pandemic has changed treatment decisions by medical staff.^14^, ^16^

This study was conducted in Belgium, starting from the first wave of the pandemic. To explain, the pandemic peak in Belgium occurred at the beginning of April 2020 and, at the time of the survey, the country was in lockdown, which started on March 18. Concerning the local hospital situation, the first COVID‐19 patient was diagnosed on March 1. The hospital emergency plan, which imposed the interruption of all non‐urgent clinical activities, the reduction of the number of outpatient consultations to 25%, and the prohibition of access to accompanying persons except in special situations, as well as the introduction of all hygienic and preventive measures, had been implemented from March 13. In the absence of scientific data, a shared decision procedure was performed before continuation of the anticancer treatment at the beginning of the pandemic. For the oncologists, it was important to better understand the perception of the situation by the patients. In this study, we investigated the patients' attitude towards COVID‐19 pandemic, and in particular we inquired about the patients' fear of COVID‐19 and cancer, the perception of being protected in and out the hospital by the preventive measures taken by them, the hospital staff, and their relatives, the possibility of changing treatment choices because of the pandemic, the perception of the risk of complications and death in case of infection, and the interest in taking information about COVID‐19 and cancer.

## 2 MATERIALS AND METHODS

### 2.1 Patients selection and data collection

Patients admitted for systemic treatment for solid cancers to the day care unit of the *University Hospital of Liège in Belgium* were prospectively included in the study.

Inclusion criteria were: age ≥18 years; diagnosis of solid tumor, irrespective of the stage; treatment at the day care unit between April 14 and April 30, 2020; ability to answer a written questionnaire in French language. Exclusion criteria were: access to the day care unit for reasons other than cancer treatment, treatment with oral therapies, no understanding of French language.

A 12‐item questionnaire (Supporting Information) using Likert scale was elaborated by one oncologist of the Medical Oncology Department and thereafter submitted to all authors. After revision, the final questionnaire was approved by all authors. The questionnaire was mainly based on assessing practical issues such as patients' perceptions of safety and risks related to COVID‐19, and medical choices, in view of the fact that treatment decisions in the early period of the pandemic were often reshaped according to the health emergency. The questionnaire was distributed between April 14 and April 30, 2020 (acute phase of the pandemic) and approximately 3 months later, between July 13 and August 12 (post‐acute phase of the pandemic). The first questionnaire was carried out in person (paper questionnaire self‐administered by the patients) at the time of access to the day care unit. The second questionnaire was carried out in person for patients still undergoing treatment between July and August 2020 and by phone for patients whose treatment was over. The questionnaire inquired about the fear of COVID‐19 infection (question 1), the perception to be protected in the hospital (question 2), the measures taken by the patients, the hospital, and their household to minimize the risk of infection (questions 3–5), the awareness about oncological treatment choices (questions 6, 7, 10), the risk of complications or death due to COVID‐19 (questions 8, 9), and acquisition of information about COVID‐19 and cancer (questions 11, 12).

Information about sex, age, cancer type, treatment intent, and type of treatment were collected in the medical charts.

The study was performed in compliance with the Helsinki Declaration, was approved by the local Ethics Committee with the reference number 2020/129, and all patients signed an informed consent before inclusion in the study.

### 2.2 Statistical analysis

Descriptive statistics were performed for each item. Differences in response to the second questionnaire performed in July–August 2020 from the baseline performed in April 2020 were analyzed by means of a McNemar–Bowker test. Subgroup analysis was performed for age (cut‐off 65 years old), sex (male or female), treatment intent (curative or palliative), type of treatment (chemotherapy, immunotherapy, targeted therapy, combination of two or more modalities), cancer type (breast, lung, gastrointestinal or other type). Chi‐square test was used to test the associations between answers and cancer characteristics. The unanswered questions were excluded from calculation of *p* values.

Statistical analysis was performed by means of SPSS v25 software.

## 3 RESULTS

A total of 237 patients receiving an oncological treatment for a solid tumor in the day care unit of the University Hospital of Liège in Belgium between April 14 and April 30, 2020 were included in the study, 197 of which answered a second questionnaire in July/August 2020. The median age at the time of study inclusion was 63 years (range 26–90), 61.6% (*n* = 146) of patients were female and 38.4% (n = 91) male. The treatment intent was curative for 36.7% (*n* = 87) of patients and palliative for 63.3% (*n* = 150). The type of treatment received was chemotherapy in 44.7% of the cases (*n* = 106, 47 of which with curative intent), immunotherapy in 25.3% of the cases (*n* = 60, 17 of which with curative intent), targeted therapy in 15.2% of the cases (*n* = 36, 14 of which with curative intent), and combination therapy in 14.8% of the cases (*n* = 35, nine of which with curative intent). The primary cancer sites were: lung (24.9%, *n* = 59), breast (22.8%, *n* = 54), gastrointestinal tract (19.4%, *n* = 46), and other (32.9%, *n* = 78). Details of patients' characteristics at the two timepoints are reported in Table 1.

**TABLE 1:** Patients characteristics

	First questionnaire N = 237	Second questionnaire N = 197
Age		
Median (range)	63 (26–90)	63 (26–90)
<65 years	138 (58.2%)	112 (56.9%)
≥65 years	99 (41.8%)	85 (43.1%)
Sex		
Male	91 (38.4%)	74 (37.6%)
Female	146 (61.6%)	123 (62.4%)
Treatment intent		
Curative	87 (36.7%)	74 (37.6%)
Palliative	150 (63.3%)	123 (62.4%)
Type of treatment		
Chemotherapy	106 (44.7%)	84 (42.6%)
Immunotherapy	60 (25.3%)	52 (26.4%)
Targeted therapy	36 (15.2%)	34 (17.3%)
Combination	35 (14.8%)	27 (13.7%)
Cancer site		
Lung	59 (24.9%)	52 (26.4%)
Breast	54 (22.8%)	48 (24.4%)
Ovary	16 (6.8%)	15 (7.6%)
Melanoma	15 (6.3%)	10 (5.1%)
Colon	12 (5.1%)	10 (5.1%)
Pancreas	9 (3.8%)	6 (3.0%)
Cervix	9 (3.8%)	7 (3.6%)
Stomach	8 (3.4%)	7 (3.6%)
Kidney	7 (3.0%)	5 (2.5%)
Endometrium	7 (3.0%)	7 (3.6%)
Esophagus	7 (3.0%)	4 (2.0%)
Rectum	7 (3.0%)	4 (2.0%)
Head and neck	6 (2.5%)	5 (2.5%)
Bladder	4 (1.7%)	4 (2.0%)
Glioblastoma	3 (1.3%)	3 (1.5%)
Cholangiocarcinoma	3 (1.3%)	2 (1.0%)
Prostate	3 (1.3%)	2 (1.0%)
Sarcoma	2 (0.8%)	2 (1.0%)
Other	6 (2.5%)	4 (2.0%)

Abbreviation: N, number.

Forty fewer questionnaires were collected in July/August as compared to April due to death (*n* = 10), patients' refusal (*n* = 24), and inability to reach the patients because of deteriorated clinical conditions (*n* = 6).

### 3.1 Worries about COVID‐19

**FIGURE 1:**
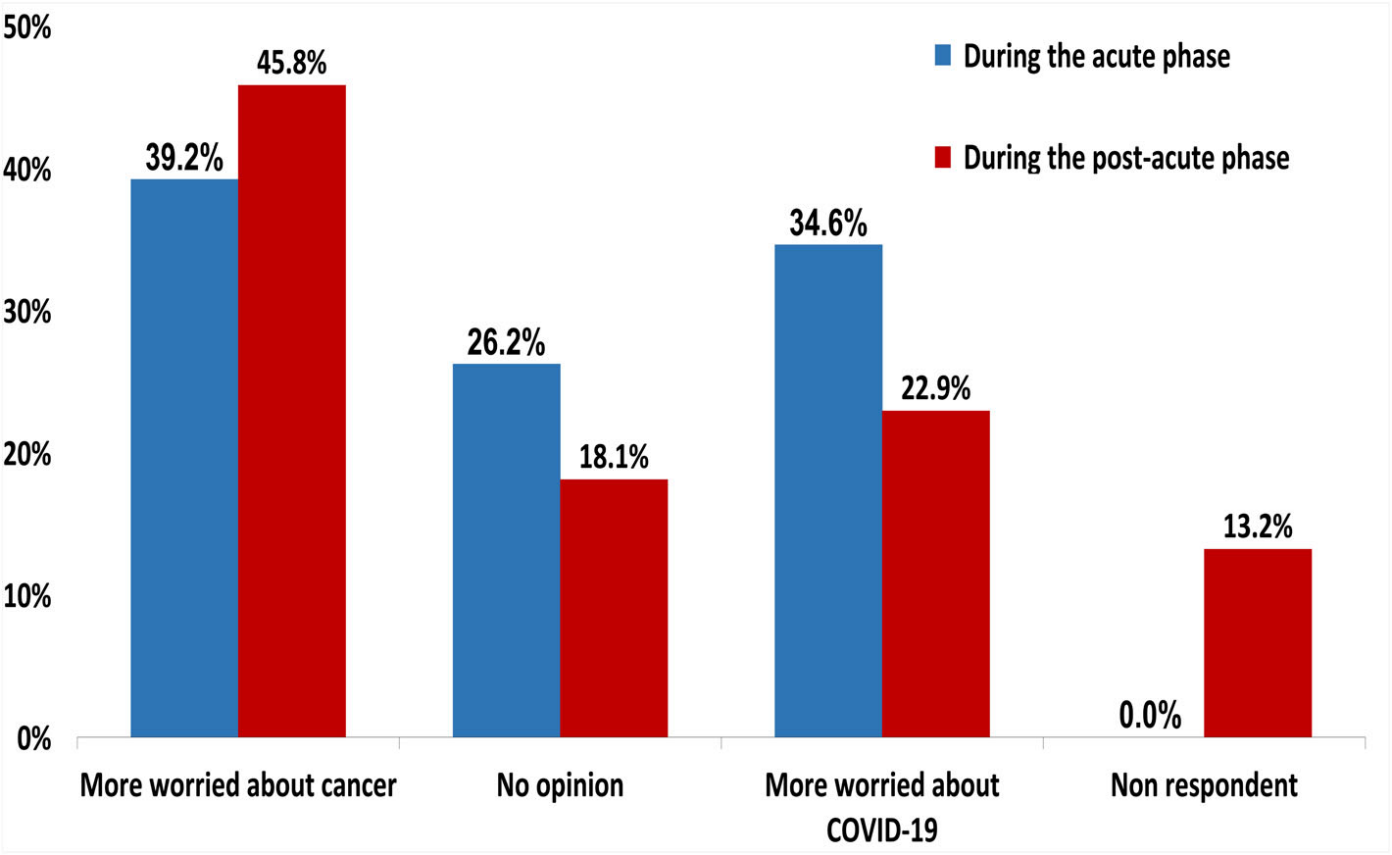
Worries about COVID‐19 and cancer. Bar graphs showing the proportion of patients more worried about COVID‐19 or cancer during the acute phase (blue) and during the post‐acute phase (red)

Patients were more worried about COVID‐19 in the acute phase compared with the post‐acute phase (34.6% vs. 22.9%), where patients were more worried about cancer (39.2% vs. 45.8% in the acute and post‐acute phase, respectively), with a statistically significant difference (*p* = .013, Figure 1).

No significant differences were observed at baseline according to age group, sex, and primary cancer site (Table S1). Patients in palliative care were more worried about cancer than COVID‐19 (40.7% vs. 28.0%, *p* = .009), while patients receiving a treatment with curative intent were more worried about COVID‐19 than cancer (46.0% vs. 36.8%, *p* = .009). Moreover, 40.6% of patients receiving chemotherapy and 41.7% of patients receiving targeted therapy were more worried about COVID‐19 than cancer in comparison with those receiving immunotherapy (28.3%) or combination therapy (20%), with differences statistically significant (*p* = .006).

During the post‐acute phase, no significant differences were observed according to sex, treatment intent, treatment type, and cancer site (Table S1). Nevertheless, patients younger than 65 years were more worried about cancer than older patients (58.9% vs. 44.7%) and less worried about COVID‐19 (19.6% vs. 35.3%, *p* = .041).

### 3.2 Security and measures to minimize the risk of infection

Most patients considered the hospital safe (Table 2) during both the acute phase (83.5%) and the post‐acute phase (91.4%), with a nonsignificant difference between the two timepoints (*p* = .081). Subgroup analysis showed significant differences during the acute phase according to age (*p* = .002), with a lower proportion of patients younger than 65 years considering the hospital safe (79.7%) than older patients (88.8%), and according to sex, with a lower proportion of women (79.3%) than men (90.1%) considering the hospital safe (*p* = .014).

**TABLE 2:** Security and measures to minimize the risk of contagion

	During the acute phase						During the post‐acute phase					
	Strongly disagree	Disagree	No opinion	Agree	Strongly agree	No response	Strongly disagree	Disagree	No opinion	Agree	Strongly agree	No response
Do you perceive the hospital safe?	1.3%	5.9%	9.3%	33.9%	49.6%	–	0.4%	3.1%	5.1%	37.1%	54.3%	–
Do you perceive your home safe and adopt all the preventive measures at home?	1.3%	1.3%	2.9%	22.1%	71.7%	0.8%	0.0%	0.0%	3.0%	22.8%	73.6%	0.5%
Do you adopt all the preventive measures outside home?	0.8%	0.4%	1.7%	21.1%	75.1%	0.8%	0.0%	0.0%	3.0%	21.3%	74.6%	1.0%
In your household/environment do everyone adopt all the preventive measures?	2.1%	1.3%	3.4%	25.7	65.8%	1.7%	0.0%	1.0%	6.6%	27.4%	64.0%	0.8%

The majority of patients perceived their home safe and adopt all the preventive measures (Table 2) during both the acute phase (93.8%) and the post‐acute phase (96.4%). No patients perceived their home as not safe in the post‐acute phase compared to 2.6% of patients during the acute phase (*p* < .0001). Subgroup analysis showed that a higher proportion of women (97.9%) perceived higher risk and took all measures to prevent infection at home than man (88.9%) did, with a significant difference during the acute phase (*p* = .047).

Likewise, a similar proportion of patients declared to adopt all the preventive measures to avoid contagion outside their home (Table 2) during the acute phase (96.2%) and the post‐acute phase (95.9%). No patient declared that preventive measures were not taken outside home during the post‐acute phase compared to 1.2% of patients during the acute phase (*p* < .0001).

Moreover, a similar proportion of patients declared that everyone in their household/environment adopt all the preventive measures (Table 2) during the acute phase (91.5%) and the post‐acute phase (91.4%), with a slightly higher proportion of patients considering that their relatives did not adopt all the preventive measures during the acute phase (3.4%,) compared to the post‐acute phase (1.0%; *p* < .0001).

### 3.3 Oncological treatment

**FIGURE 2:**
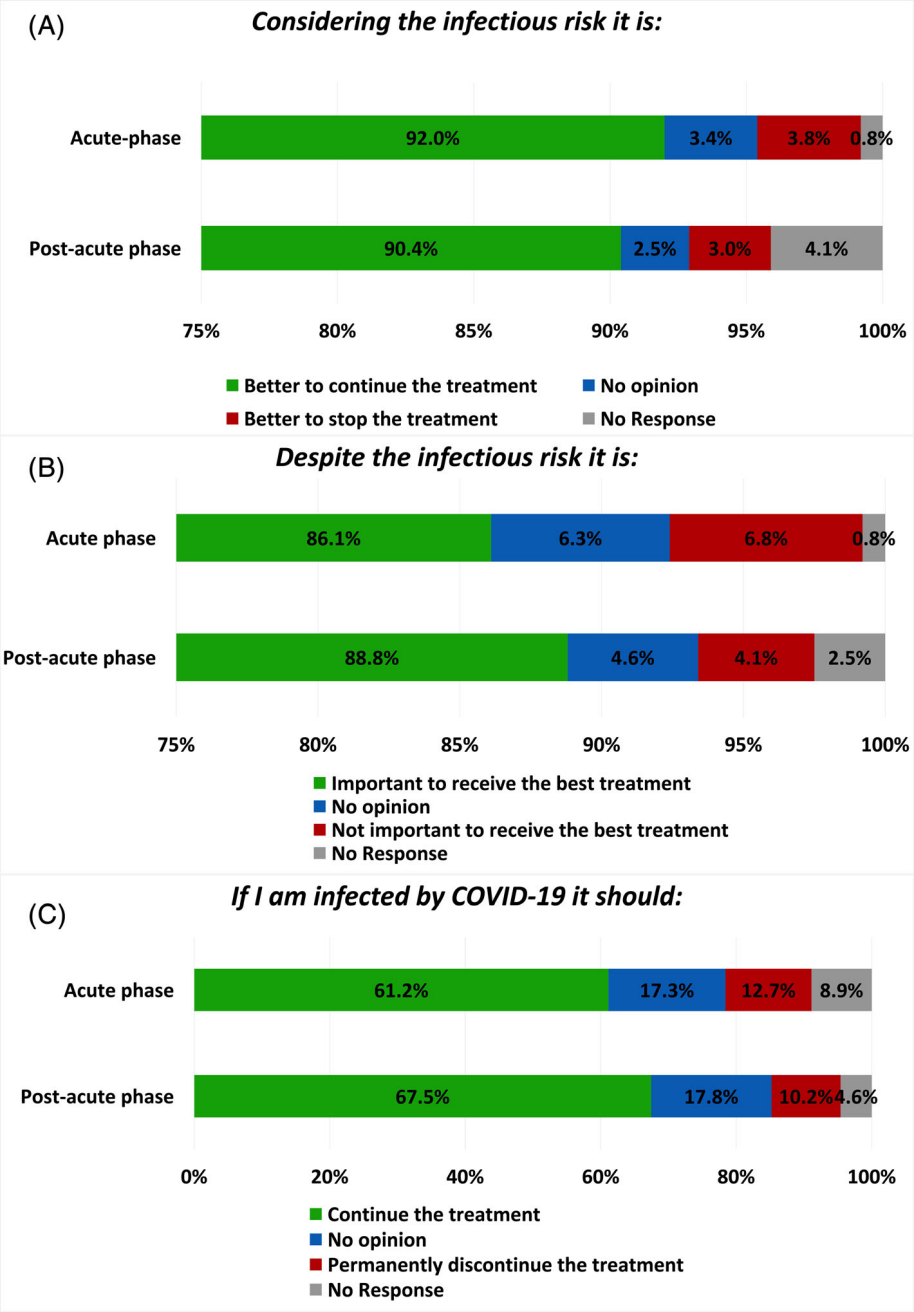
Patients' perception about oncological treatment. Bar graphs representing the patients' perception about the possibility to stop treatment due to the infectious risk (A), the importance to receive the best treatment (B), and the possibility to permanently discontinue the treatment if infected by Sars‐Cov‐2 (C)

Most patients disagreed with the possibility of stopping treatment due to infectious risk (Figure 2A) during both the acute phase (92.0%) and the post‐acute phase (90.4%), without significant differences between the two timepoints (*p* = .707) and across the various subgroups (Table S2). Moreover, 86.1% of the patients declared that it is important to receive the best treatment during the acute phase and 88.8% during the post‐acute phase (*p* = .266, Figure 2B). No significant differences were observed across subgroups (Table S3).

In case of infection, 12.7% of patients during the acute phase and 10.2% during the post‐acute phase declared that the oncological treatment should be definitely discontinued (Figure 2C), with no significant difference between the two timepoints (*p* = 0.812). No differences were observed according to patients' characteristics (Table S4). During the post‐acute phase, a higher proportion of men than women thought that treatment should be permanently discontinued if infected by SARS‐CoV‐2 (16.4% vs. 7.0%, *p* = .043).

### 3.4 Risk of complications and death

**FIGURE 3:**
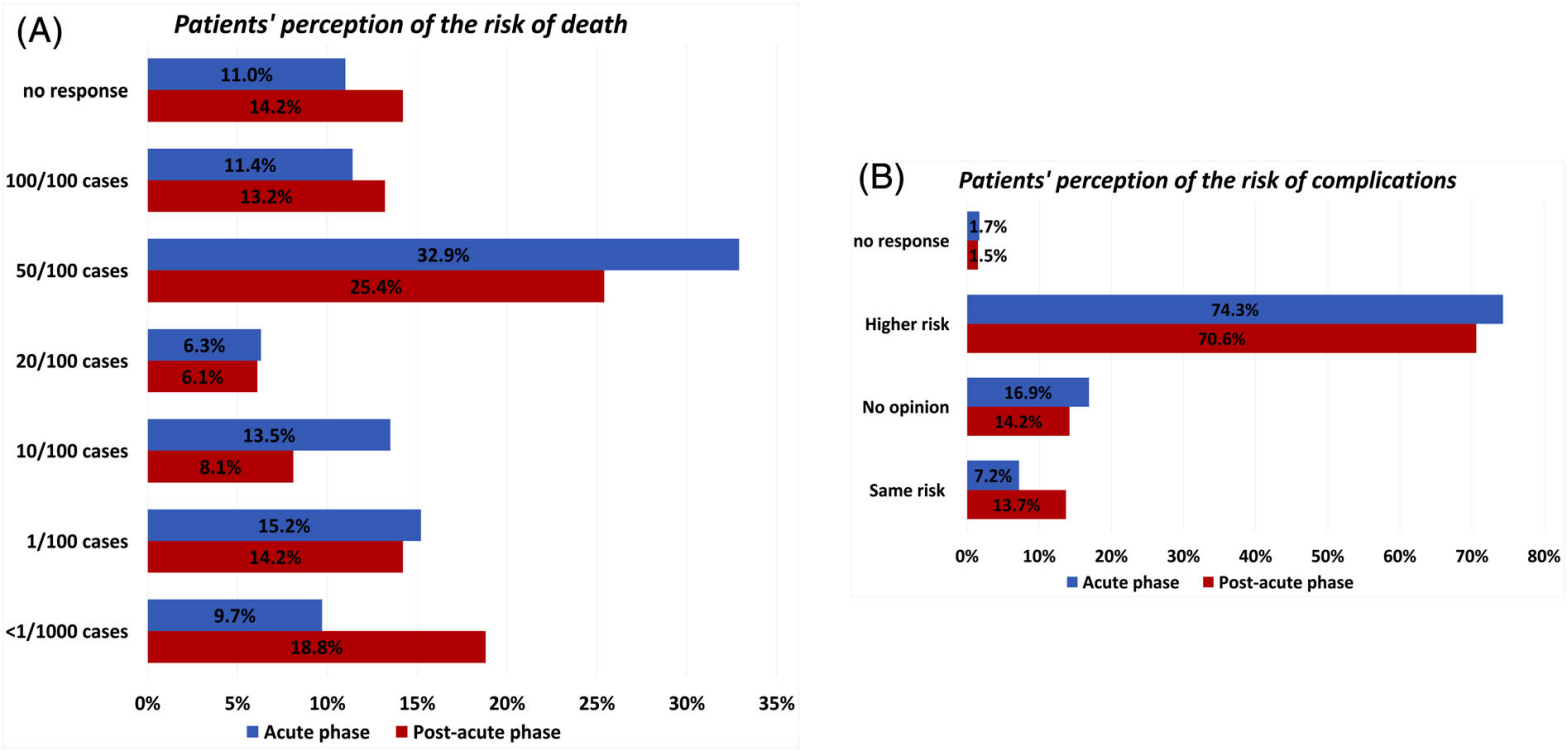
Risk of death and complications by COVID‐19. Bar graphs representing patients' perception of the risk of death (A) and complications (B) if infected by Sars‐Cov‐2 during the acute phase (blue) and the post‐acute phase (red)

The majority of cancer patients were aware of belonging to a high‐risk category, with the majority stating to have a death risk of approximately 50% if infected by SARS‐CoV‐2 (Figure 3A). There were no statistically significant differences in the perception of the risk of death during the acute and the post‐acute phase (*p* = .138). A higher proportion of lung cancer patients estimated their risk of death to be approximately 100% compared to other cancer sites (34.1% for lung cancer vs. 15.4% for breast cancer, 7.1% for gastrointestinal cancer, and 5.2% for other cancer sites, *p* = .007, Table S5).

Moreover, the majority of cancer patients mentioned that they were at higher risk to develop complications than the general population (Figure 3B), in particular 74.3% during the acute phase and 70.6% during the post‐acute phase said they had a higher risk, while 7.2% (*n* = 17/237) and 13.7% (*n* = 27/197), respectively, said they had the same risk (*p* = 0.030). No differences were observed according to patients' characteristics (Table S6). A lower proportion of patients receiving immunotherapy in the post‐acute phase thought they were at higher risk of complications than patients receiving other treatments (53.8% for immunotherapy vs. 79.8% for chemotherapy vs. 71.0% for targeted therapy vs. 81.5% for combination therapy, *p* = .047). A higher proportion of lung cancer patients thought they were at higher risk of complications during the acute phase of the pandemic (86% of lung cancer patients vs. 72.2% for breast, 82.6% for gastrointestinal and 65.8% for other cancer sites, *p* = .015).

### 3.5 Information about COVID‐19 and cancer

**FIGURE 4:**
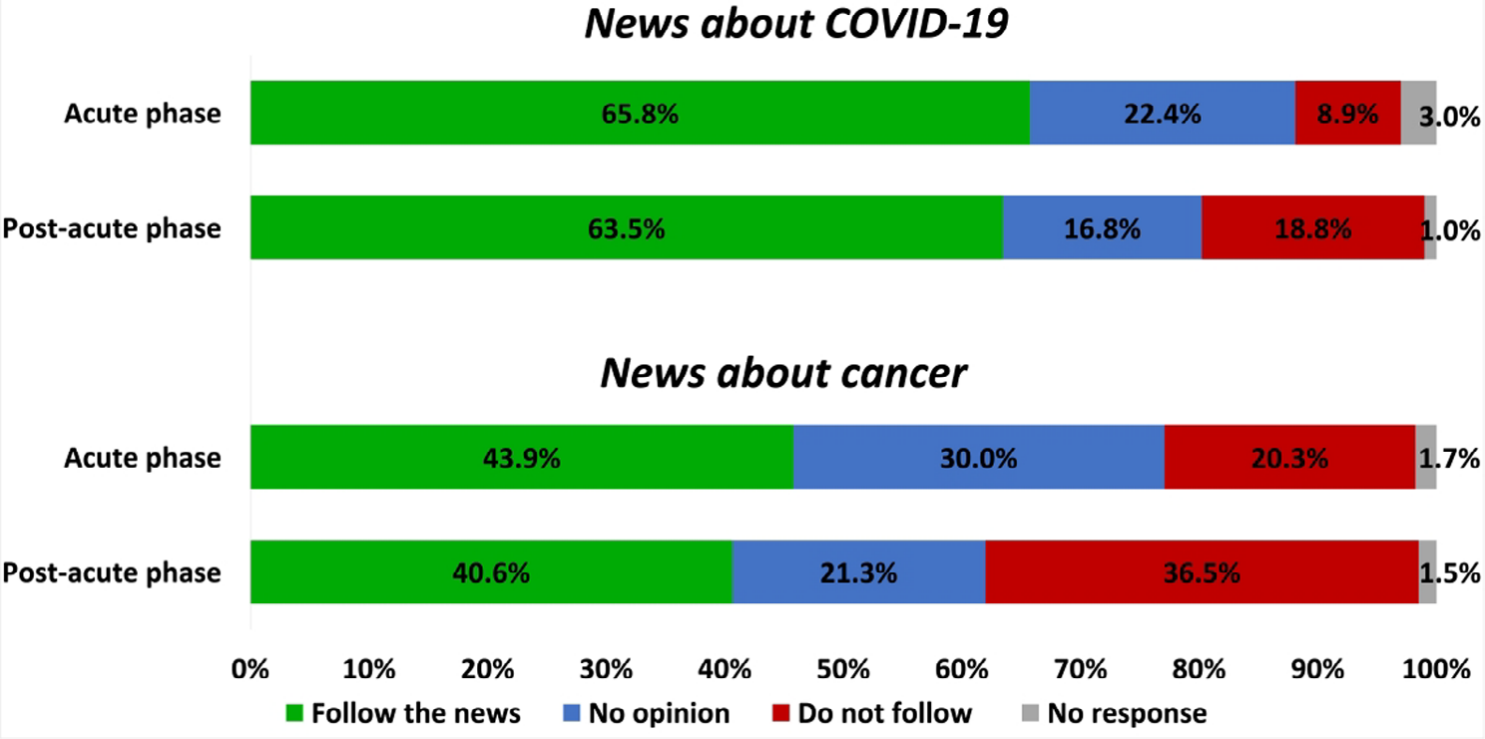
Interest in following the news about COVID‐19 and cancer. Bar graphs representing the proportion of patients following the news about COVID‐19 and cancer

Most patients declared to follow news about COVID‐19 in both the acute phase (65.8%) and post‐acute phase (63.5%), while a doubled proportion of patients did not follow the news about COVID‐19 during the post‐acute phase (8.9% in the acute phase vs. 18.8% in the post‐acute phase; *p* = .034; Figure 4). A lower proportion of patients declared to follow news about cancer compared to COVID‐19. The higher interest in following news about COVID‐19 than cancer was significant in both the acute phase (*p* < 0.0001) and the post‐acute phase (*p* < 0.0001). A higher proportion of women followed news about cancer compared to men (50% vs. 36%, respectively; *p* = 0.014).

## 4 DISCUSSION

The advent of the COVID‐19 pandemic has led to substantial reassessment of social relationship and work activities, in addition to hospital activities. This has led to a high level of psychological distress among the general population, health workers, and patients with chronic diseases, such as cancer.^2^, ^17^, ^18^, ^19^ Indeed, cancer patients are frail, due to comorbidity and treatment, and they need continuous care, which often cannot be postponed, despite the overloading of hospitals and the subsequent difficulty in maintaining nonurgent medical activities. The fear to get infected and the social distancing measures induced a deterioration of well‐being of this subgroup of patients. Stressful conditions are known to induce a worsening of chronic diseases through the activation of a neuroendocrine axis with the production of immunomodulatory cytokines, which ultimately lead to disruption of immunosurveillance.^20^ Concerning the specific context of oncological diseases, an association between a worse outcome and loneliness has been observed.^21^

When considering the answers given to the two questionnaires, the different timing of the epidemic must be taken into account. In fact, the first questionnaire was filled in during the first wave of the outbreak, when hospitals were overloaded with COVID‐19 patients, hospital access restrictions were imposed at local level, and a lockdown was active at national level. The second questionnaire was filled in when the load of infected patients was significantly reduced, hospital activity had returned to normal and at national level lockdown was replaced by measures of social distancing, reduction in the number of interpersonal contacts, and mask wearing required in public places. Besides the different national regulations aimed at reducing contact and the risk of infection and the different number of positive cases at the time the questionnaire was administered, there was also an improved knowledge of the disease between the two phases in which the study was performed. More and more appropriate guidelines for the prevention and treatment of the disease were established and the level of information was increasing. These aspects probably contributed to the responses given by patients, in particular both the awareness of improved treatment and the reduction in cases led patients to be more afraid by the tumor than by the COVID‐19 in the second phase of the study.

In our study, patients showed a higher level of concern for COVID‐19 than for cancer, mainly if older, receiving a treatment with curative intent, chemo‐ or targeted therapy. The majority of patients treated with immunotherapy were more concerned about cancer than about COVID‐19. Only 71.4% of patients receiving immunotherapy thought they were at greater risk of complications if infected by SARS‐CoV‐2 compared to 81.1% of patients treated with chemotherapy. Our study showed the lack of awareness among patients receiving immunotherapy, who thought they were at lower risk of complications by COVID‐19 infection compared to patients receiving chemotherapy, whereas it was the contrary. In fact, a previously published study demonstrated that treatment with immunotherapy was predictive of a more severe disease, whereas chemotherapy was not.^22^ One possible explanation for this attitude could be related to the type of treatment, which leads patients to think that their immune system is stimulated in general, and therefore also against infections. The stage of the disease could be a possible explanation for their greater concern with cancer than with COVID‐19, considering that most patients receive immunotherapy in the palliative setting. In this context, we must consider that in our study about one third of patients received immunotherapy in the adjuvant setting.

All patients showed awareness to be at higher risk for death and complications than the general population. These concerns are likely to prompt cancer patients to take all necessary precautions to avoid infection. The same observation has been previously reported in another study, where cancer patients followed hygiene requirements and avoided public spaces more frequently than healthy subjects.^19^ It is interesting to note that patients felt safe in our hospital, where all the necessary hygiene measures were taken. A direct consequence of the extreme attention paid in our oncology department and by patients in complying preventive measures is the low infection rate recorded among these patients, which, although slightly higher than in the general population, remains lower than expected.^23^, ^24^

Remarkably, although cancer patients are scared of COVID‐19 and aware that they are at greater risk of complications, they still expect to receive the best oncological treatment and have not considered the possibility of discontinuing or postponing treatment due to the pandemic. This is in contrast with what was done in most cancer units, where treatment was modulated to minimize the number of hospital accesses and palliative treatment was more frequently discontinued in the final stages of disease.^9^ This could lead to problems in the physician‐patient relationship, as well to legal issues. In this context, psychological support, optimal communication, and sharing of decisions with the patient could help overcome this issue.^25^

Patients acknowledged they were following all the news related to COVID‐19 closely more than for cancer. This response was expected, considering the huge mediatic resonance of the COVID‐19 outbreak. Neither the type of information nor the authenticity of the sources used by the patients to inform themselves was investigated, but a subjective question was asked with the only aim of investigating whether patients were more likely to inform themselves about COVID‐19 or cancer. On the other hand, since the start of the pandemic, the accessibility to the relevant information on COVID‐19 has always been predominant, so the ease of access to these data has certainly contributed to a greater interest about COVID‐19 than about cancer. Although the answers reflect patients' involvement in following actuality, a previous study showed that hyperinformation can lead to a higher level of psychological distress.^2^

The limitations of this study are its monocentricity, the wide heterogeneity of the study population (primary cancer site, treatment intent, treatment type, and patients' age), the short follow up limited to 3 months, the failure to study the association between distress level and outcome, the lack of administration of validated questionnaires to assess distress level and psychological change due to the pandemic, the absence of a validation of the questionnaire administered. However, the strength of this study is its focus on the practical aspects of pandemic management from the patient's point of view, highlighting critical issues such as the difficult acceptance of treatment interruption or modification due to the health crisis.

In conclusion, the majority of patients stated that they worried about COVID‐19, and therefore preventive measures to avoid contagion were taken very carefully. Although most of them were aware of their greater risk of complications than general population, patients were not aware of the possibility of adapting cancer treatments due to the pandemic. These observations lead to an important consideration regarding both the risk of undertreatment and the acceptance of treatment adaptation by patients. Greater attention to the psychological aspect of the patient and better communication are needed to optimize the current and potential management of future health emergencies.

## CONFLICT OF INTEREST

Dr. B.S. reports consulting or Advisory role for Clovis Oncology, Astellas, Janssen, and Sanofi and received financial support for travel and/or accommodation from Janssen, outside the submitted work. Dr. A.S. reports Advisory board role for MSD, AstraZeneca, BMS, Boehringer‐Ingelheim, Roche, Takeda, and Pfizer, outside the submitted work. Dr. G.J. reports grants, personal fees, and nonfinancial support from Novartis, Roche, Pfizer; personal fees and nonfinancial support from Lilly, Amgen, BMS, and Astra‐Zeneca; personal fees from Daiichi Sankyo and Abbvie; nonfinancial support from Medimmune and MerckKGaA, outside the submitted work. The other authors have nothing to disclose.

## AUTHOR CONTRIBUTIONS

All authors had full access to the data in the study and take responsibility for the integrity of the data and the accuracy of the data analysis. *Conceptualization*: C.E.O., H.S., A.R., B.S., E.G., A.C., L.N., O.W., A.C., L.L., J.C., C.G., P.F., N.M., A.P., G.J.; *Data curation*: C.E.O., H.S., A.C., L.N., O.W., A.C., G.J.; *Formal analysis*: C.E.O., H.S., G.J.; *Funding acquisition*: H.S., G.J.; *Investigation*: C.E.O., H.S., A.R., B.S., M.L., M.G., E.G., A.C., L.N., O.W., A.C., C.L., A.P., A.S., L.L., F.T., J.C., C.G., P.F., M.P., B.D., F.V., N.M., A.P., G.J.; *Methodology*: C.E.O., H.S., A.R., B.S., M.L., M.G., E.G., A.C., L.N., O.W., A.C., C.L., A.P., A.S., L.L., F.T., J.C., C.G., P.F., M.P., B.D., F.V., N.M., A.P., G.J.; *Project administration*: H.S., G.J.; *Resources*: C.E.O., H.S., G.J.; *Software*: C.E.O.; *Supervision*: G.J.; *Validation*: C.E.O., H.S., A.R., B.S., M.L., M.G., E.G., A.C., L.N., O.W., A.C., C.L., A.P., A.S., L.L., F.T., J.C., C.G., P.F., M.P., B.D., F.V., N.M., A.P., G.J.; *Writing – original draft*: C.E.O., G.J.; *Writing – review and editing*: C.E.O., H.S., A.R., B.S., M.L., M.G., E.G., A.C., L.N., O.W., A.C., C.L., A.P., A.S., L.L., F.T., J.C., C.G., P.F., M.P ., B.D., F.V., N.M., A.P., G.J.

## ETHICAL STATEMENT

This project was approved by the local Ethics Committee with the reference number 2020/129. All patients signed an informed consent before inclusion in the study.

## Supporting information


**Appendix** S1. Supporting Information

## Data Availability

Data are available upon appropriate request to the corresponding author.
