# Supplementary material for: Oncological patients' reactions to COVID‐19 pandemic: A single institution prospective study
Source: Cancer Rep (Hoboken). 2021 Oct 12;5(10):e1571. doi: 10.1002/cnr2.1571 (PMC8646835; doi:10.1002/cnr2.1571)
Supplement: Supplementary file 1 — Appendix S1. Supporting Information [file CNR2-5-e1571-s001.pdf]

Supplementary materials

Survey 2
Table S1. Subgroups analysis concerning worries about COVID-19 and cancer. 5
Table S2. Subgroups analysis concerning the possibility to stop the treatment considering the risk of Sars-Cov-2 infection. 6
Table S3. Subgroups analysis concerning the importance to receive the better treatment despite the risk of infection. 7
Table S4. Subgroups analysis concerning the possibility to discontinue the treatment if infected by Sars-Cov-2. 8
Table S5. Subgroups analysis concerning the awareness of the risk of death. 9
Table S6. Subgroups analysis concerning the awareness of the risk of complications. 10

Survey

Study name: Study of the fears and perceptions of patients with a solid tumour in relation to the pandemic COVID-19

Name:	Surname:
Date of birth:	
Sex:	
Date of informed consent:	
Date of questionnaire:	

Cancer type:

Type of treatment: ☐ Chemotherapy ☐ Immunotherapy ☐ Targeted therapy

Drugs received:

For the questions asked below, please answer as follows. On an evaluation scale from 1 to 5, 1 being "strongly disagree" and 5 being "strongly agree" with the proposed sentence.
1 Strongly disagree
2 No agreement
3 Without an opinion, it makes no difference to me
4 Agreed
5 Strongly agree

1. I am more concerned about the risk of catching Covid-19 (coronavirus) than about the health problems related to the cancer:

Strongly disagree 1 2 3 4 5 Strongly agree

2. I feel safe in the hospital, all measures to minimize the risk of infection are taken:

Strongly disagree 1 2 3 4 5 Strongly agree

3. I take all the necessary measures at home to minimize the risk of infection:

Strongly disagree 1 2 3 4 5 Strongly agree

4. I take outside home (going to the hospital, to do shopping, to go to work...) all measures to minimize the risk of infection:

Strongly disagree 1 2 3 4 5 Strongly agree

5. Everyone in my household/environment is taking every measure to minimize the risk of infection:

Strongly disagree 1 2 3 4 5 Strongly agree

6. I ask myself if it would not be better to stop treatment because of the epidemic:

Strongly disagree 1 2 3 4 5 Strongly agree

7. Despite the current epidemic, it is important that I receive the best treatment, even if it increases the risk of infection:

Strongly disagree 1 2 3 4 5 Strongly agree

8. If I am infected with the the SARS-CoV-2 I am concerned that my risk of dying from it is:

< 1/1000	1/100	10/100	20/100	50/100	100/100

9. If I am infected by SARS-CoV-2, I am more at risk of developing greater complications or symptoms than people who do not have cancer:

Strongly disagree	1	2	3	4	5	Strongly agree

10. If I am infected by SARS-CoV-2, I will need to permanently discontinue cancer treatment:

Strongly disagree	1	2	3	4	5	Strongly agree

11. I follow very closely all the news about Covid-19 (TV, internet, reading...):

Strongly disagree	1	2	3	4	5	Strongly agree

12. I follow very closely all the news about cancer (TV, internet, reading...):

Strongly disagree	1	2	3	4	5	Strongly agree

Table S1. Subgroups analysis concerning worries about COVID-19 and cancer.

		Most worried by cancer N (%)	*No opinion N (%)*	*Most worried by COVID-19 N (%)*	*p value*
During the acute phase	***Age***				
	< 65 y	58 (42%)	36 (26.1%)	44 (31.9%)	0.507
	≥ 65 y	35 (35.4%)	26 (26.3%)	38 (38.4%)	
	Sex				
	Male	39 (42.9%)	22 (24.2%)	30 (33%)	0.661
	Female	54 (37%)	40 (27.4%)	52 (35.6%)	
	Treatment intent				
	Curative	32 (36.8%)	15 (17.2%)	40 (46%)	**0.009**
	Palliative	61 (40.7%)	47 (31.3%)	42 (28%)	
	Type of treatment				
	Chemotherapy	42 (39.6%)	21 (19.8%)	43 (40.6%)	**0.006**
	Immunotherapy	30 (50%)	13 (21.7%)	17 (28.3%)	
	Targeted therapy	6 (16.7%)	15 (41.7%)	15 (41.7%)	
	Combination	15 (42.9%)	13 (37.1%)	7 (20%)	
	Cancer site				
	Breast	13 (24.1%)	17 (31.5%)	24 (44.4%)	0.166
	Lung	27 (45.8%)	13 (22%)	19 (32.2%)	
	Gastrointestinal	16 (34.8%)	14 (30.4%)	16 (34.8%)	
	Other	37 (47.4%)	18 (23.1%)	23 (29.5%)	
During the post-acute phase	***Age***				
	< 65 y	66 (58.9%)	24 (21.4%)	22 (19.6%)	**0.041**
	≥ 65 y	38 (44.7%)	17 (20%)	30 (35.3%)	
	Sex				
	Male	36 (48.6%)	17 (23%)	21 (28.4%)	0.661
	Female	68 (55.3%)	24 (19.5%)	31 (25.2%)	
	Treatment intent				
	Curative	43 (58.1%)	14 (18.9%)	17 (23%)	0.530
	Palliative	61 (49.6%)	27 (22%)	35 (28.5%)	
	Type of treatment				
	Chemotherapy	44 (52.4%)	18 (21.4%)	22 (26.2%)	0.316
	Immunotherapy	32 (61.5%)	10 (19.2%)	10 (19.2%)	
	Targeted therapy	15 (44.1%)	5 (14.7%)	14 (41.2%)	
	Combination	13 (48.1%)	8 (29.6%)	6 (22.2%)	
	Cancer site				
	Breast	28 (58.3%)	7 (14.6%)	13 (27.1%)	0.453
	Lung	26 (50%)	11 (21.2%)	15 (28.8%	
	Gastrointestinal	13 (40.6%)	7 (21.9%)	12 (37.5%)	
	Other	37 (52.8%)	16 (24.6%)	12 (18.5%)	

Table S2. Subgroups analysis concerning the possibility to stop the treatment considering the risk of SARS-CoV-2 infection.

		Better to stop the treatment N (%)	No opinion N (%)	Better to continue the treatment N (%)	p value
During the acute phase	***Age***				
	< 65 y	7 (5.1%)	8 (5.8%)	122 (89.1%)	0.420
	≥ 65 y	9 (9.2%)	7 (7.1%)	82 (83.7%)	
	Sex				
	Male	4 (4.4%)	5 (5.5%)	82 (90.1%)	0.440
	Female	12 (8.3%)	10 (6.9%)	122 (84.7%)	
	Treatment intent				
	Curative	7 (8.1%)	4 (4.7%)	75 (87.2%)	0.609
	Palliative	9 (6%)	11 (7.4%)	129 (86.6%)	
	Type of treatment				
	Chemotherapy	7 (6.6%)	9 (8.5%)	90 (84.9%)	0.773
	Immunotherapy	3 (5.1%)	3 (5.1%)	53 (89.8%)	
	Targeted therapy	4 (11.4%)	2 (5.7%)	29 (82.9%)	
	Combination	2 (5.7%)	1 (2.9%)	32 (91.4%)	
	Cancer site				
	Breast	4 (7.5%)	2 (3.8%)	47 (88.7%)	0.082
	Lung	3 (5.2%)	4 (6.9%)	51 (87.9%)	
	Gastrointestinal	1 (2.5%)	7 (15.2%)	38 (82.6%)	
	Other	8 (10.3%)	2 (2.6%)	68 (87.2%)	
During the post-acute phase	***Age***				
	< 65 y	5 (4.5%)	5 (4.5%)	100 (90.9%)	0.951
	≥ 65 y	3 (3.7%)	4 (4.9%)	75 (91.5%)	
	Sex				
	Male	2 (2.7%)	6 (8.2%)	65 (89%	0.152
	Female	6 (5%)	3 (2.5%)	110 (92.4%)	
	Treatment intent				
	Curative	1 (1.4%)	2 (2.8%)	69 (95.8%)	0.191
	Palliative	7 (5.8%)	7 (5.8%)	106 (88.3%)	
	Type of treatment				
	Chemotherapy	4 (4.9%)	3 (3.7%)	75 (91.5%)	0.885
	Immunotherapy	3 (5.8%)	3 (5.8%)	46 (88.5%)	
	Targeted therapy	0 (0%)	2 (6.3%)	30 (93.8%)	
	Combination	1 (3.8%)	1 (3.8%)	24 (92.3%)	
	Cancer site				
	Breast	0 (0%)	2 (4.5%)	42 (95.5%)	0.510
	Lung	3 (5.8%)	4 (7.7%)	45 (86.5%)	
	Gastrointestinal	2 (6.3%)	0 (0%)	30 (93.8%)	
	Other	3 (4.7%)	3 (4.7%)	58 (90.6%)	

Table S3. Subgroups analysis concerning the importance to receive the better treatment despite the risk of infection.

		Not important to receive the better treatment N (%)	No opinion N (%)	Important to receive the better treatment N (%)	p value
During the acute phase	***Age***				
	< 65 y	7 (5.1%)	8 (5.8%)	122 (89.1%)	0.420
	≥ 65 y	9 (9.2%)	7 (7.1%)	82 (83.7%)	
	Sex				
	Male	4 (4.4%)	5 (5.5%)	82 (90.1%)	0.440
	Female	12 (8.3%)	10 (6.9%)	122 (84.7%)	
	Treatment intent				
	Curative	7 (8.1%)	4 (4.7%)	75 (87.2%	0.609
	Palliative	9 (6%)	11 (7.4%)	129 (86.6%)	
	Type of treatment				
	Chemotherapy	7 (6.6%)	9 (8.5%)	90 (84.9%)	0.773
	Immunotherapy	3 (5.1%)	3 (5.1%)	53 (89.8%)	
	Targeted therapy	4 (11.4%)	2 (5.7%)	29 (82.9%)	
	Combination	2 (5.7%)	1 (2.9%)	32 (91.4%)	
	Cancer site				
	Breast	4 (7.5%)	2 (3.8%)	47 (88.7%)	0.082
	Lung	3 (5.2%)	4 (6.9%)	51 (87.9%)	
	Gastrointestinal	1 (2.2%)	7 (15.2%)	38 (82.6%)	
	Other	8 (10.3%)	2 (2.6%)	68 (87.2%)	
During the post-acute phase	***Age***				
	< 65 y	5 (4.5%)	5 (4.5%)	100 (90.9%)	0.951
	≥ 65 y	3 (3.7%)	4 (4.9%)	75 (91.5%)	
	Sex				
	Male	2 (2.7%)	6 (8.2%)	65 (89%)	0.152
	Female	6 (5%)	3 (2.5%)	110 (92.4%)	
	Treatment intent				
	Curative	1 (1.4%)	2 (2.8%)	69 (95.8%)	0.191
	Palliative	7 (5.8%)	7 (5.8%)	106 (88.3%)	
	Type of treatment				
	Chemotherapy	4 (4.9%)	3 (3.7%)	75 (91.5%)	À.885
	Immunotherapy	3 (5.8%)	3 (5.8%)	46 (88.5%)	
	Targeted therapy	0 (0%)	2 (6.3%)	30 (93.8%)	
	Combination	1 (3.8%)	1 (3.8%)	24 (92.3%)	
	Cancer site				
	Breast	0 (0%)	2 (4.5%)	42 (95.5%)	0.510
	Lung	3 (5.8%)	4 (7.7%)	45 (86.5%)	
	Gastrointestinal	2 (6.3%)	0 (0%)	30 (93.8%)	
	Other	3 (4.7%)	3 (4.7%)	58 (90.6%)	

Table S4. Subgroups analysis concerning the possibility to discontinue the treatment if infected by SARS-CoV-2.

		Continue the treatment N (%)	*No opinion N (%)*	*Better to continue the treatment N (%)*	*p value*
**During the acute phase**	***Age***				
	< 65 y	82 (66.1%)	24 (19.4%)	18 (14.5%)	0.929
	≥ 65 y	63 568.5%)	17 (18.5%)	12 (13%)	
	Sex				
	Male	58 (68.2%)	16 (18.8%)	11 (12.9%)	0.943
	Female	87 (66.4%)	25 (19.1%)	19 (14.5%)	
	Treatment intent				
	Curative	57 (68.7%)	14 (16.9%)	12 (14.5%)	0.820
	Palliative	88 (66.2%)	27 (20.3%)	18 (13.5%)	
	Type of treatment				
	Chemotherapy	60 (59.4%)	22 (21.8%)	19 (18.8%)	0.344
	Immunotherapy	38 (71.7%)	9 (17%)	6 (11.3%)	
	Targeted therapy	25 (80.6%)	4 (12.9%)	2 (6.5%)	
	Combination	22 (71%)	6 (19.4%)	3 (9.7%)	
	Cancer site				
	Breast	32 (64%)	9 (18%)	9 (18%)	0.287
	Lung	37 (68.5%)	9 (16.7%)	8 (14.8%)	
	Gastrointestinal	23 (54.8%)	13 (31%)	6 (14.3%)	
	Other	53 (75.7%)	10 (14.3%)	7 (10%)	
**During the post-acute phase**	***Age***				
	< 65 y	76 (70.4%)	18 (16.7%)	14 (13%)	0.404
	≥ 65 y	57 (71.3%)	17 (21.3%)	6 (7.5%)	
	Sex				
	Male	52 (71.2%)	9 (12.3%)	12 (16.4%)	**0.043**
	Female	81 (70.4%)	26 (22.6%)	8 (7%)	
	Treatment intent				
	Curative	49 (70%)	14 (20%)	7 (10%)	0.920
	Palliative	84 (71.2%)	21 (17.8%)	13 (11%)	
	Type of treatment				
	Chemotherapy	57 (70.4%)	13 (16%)	11 (13.6%)	0.726
	Immunotherapy	35 (70%)	11 (22%)	4 (8%)	
	Targeted therapy	24 (77.4%)	4 (12.9%)	3 (9.7%)	
	Combination	17 (65.4%)	7 (26.9%)	2 (7.7%)	
	Cancer site				
	Breast	37 (84.1%)	5 (11.4%)	2 (4.5%)	0.268
	Lung	34 (68%)	10 (20%)	6 (12%)	
	Gastrointestinal	21 (65.6%)	5 (15.6%)	6 (18.8%)	
	Other	41 (66.1%)	15 (24.2%)	6 (9.7%)	

Table S5. Subgroups analysis concerning the awareness of the risk of death.

			<1/1000 N (%)	1/100 N (%)	10/100 N (%)	20/100 N (%)	50/100 N (%)	100/100 N (%)	p value
During the acute phase	*Age*								
		< 65 y	15 (12.6%)	19 (16%)	18 (15.1%)	9 (7.6%)	45 (37.8%)	13 (10.9%)	0.876
		≥ 65 y	8 (8.7%)	17 (18.5%)	14 (15.2%)	6 (6.5%)	33 (35.9%)	14 (15.2%)	
	Sex								
		Male	10 (11.9%)	14 (16.7%)	15 (17.9%)	7 (8.3%)	28 (33.3%)	10 (11.9%)	0.890
		Female	13 (10.2%)	22 (17.3%)	17 (13.4%)	8 (6.3%)	50 (39.4%)	17 (13.4%)	
	Treatment intent								
		Curative	11 (14.1%)	12 (15.4%)	13 (16.7%)	7 (9%)	27 (34.6%)	8 (10.3%)	0.705
		Palliative	12 (9%)	24 (18%)	19 (14.3%)	8 (6%)	51 (38.3%)	19 (14.3%)	
	Type of treatment								
		Chemotherapy	13 (13.5%)	10 (10.4%)	17 (17.7%)	11 (11.5%)	34 (35.4%)	11 (11.5%)	0.200
		Immunotherapy	5 (9.4%)	14 (26.4%)	7 (13.2%)	1 (1.9%)	18 (34%)	8 (15.1%)	
		Targeted therapy	2 (6.1%)	6 (18.2%)	6 (18.2%)	3 (9.1%)	14 (42.4%)	2 (6.1%)	
		Combination	3 (10.3%)	6 (20.7%)	2 (6.9%)	0 (0%)	12 (41.4%)	6 (20.7%)	
	Cancer site								
		Breast	5 (10.4%)	8 (16.7%)	8 (16.7%)	5 (10.4%)	18 (37.5%)	4 (8.3%)	0.117
		Lung	3 (5.8%)	12 (23.1%)	5 (9.6%)	1 (1.9%)	19 (36.5%)	12 (23.1%)	
		Gastrointestinal	4 (10%)	3 (7.5%)	11 (27.5%)	2 (5%)	15 (37.5%)	5 (12.5%)	
		Other	11 (15.5%)	13 (18.3%)	8 (11.3%)	7 (9.9%)	26 (36.6%)	6 (8.5%)	
During the post-acute phase	*Age*								
		< 65 y	22 (24.2%)	15 (15.3%)	12 (12.2%)	9 (9.2%)	29 (29.6%)	11 (11.2%)	0.287
		≥ 65 y	15 (21.1%)	13 (18.3%)	4 (5.6%)	3 (4.2%)	21 (29.6%)	15 (21.1%)	
	Sex								
		Male	11 (16.7%)	11 (16.7%)	6 (9.1%)	4 (6.1%)	20 (30.3%)	14 (21.2%)	0.558
		Female	26 (25.2%)	17 (16.5%)	10 (9.7%)	8 (7.8%)	30 (29.1%)	12 (11.7%)	
	Treatment intent								
		Curative	17 (27%)	11 (17.5%)	7 (11.1%)	3 (4.8%)	21 (33.3%)	4 (6.3%)	0.151
		Palliative	20 (18.9%)	17 (16%)	9 (8.5%)	9 (8.5%)	29 (27.4%)	22 (20.8%)	
	Type of treatment								
		Chemotherapy	17 (21.5%)	15 (19%)	8 (10.1%)	5 (6.3%)	25 (31.6%)	9 (11.4%)	0.857
		Immunotherapy	9 (20.9%)	8 (18.6%)	4 (9.3%)	2 (4.7%)	10 (23.3%)	10 (23.3%)	
		Targeted therapy	7 (30.4%)	2 (8.7%)	2 (8.7%)	3 (13%)	5 (21.7%)	4 (17.4%)	
		Combination	4 (16.7%)	3 (12.5%)	2 (8.3%)	2 (8.3%)	10 (41.7%)	3 (12.5%)	
	Cancer site								
		Breast	10 (25.6%)	1 (2.6%)	6 (15.4%)	4 (10.3%)	12 (30.8%)	6 (15.4%)	**0.007**
		Lung	6 (13.6%)	7 (15.9%)	3 (6.8%)	1 (2.3%)	12 (27.3%)	15 (34.1%)	
		Gastrointestinal	5 (17.9%)	7 (25%)	3 (10.7%)	4 (14.3%)	7 (25%)	2 (7.1%)	
		Other	16 (27.6%)	13 (22.4%)	4 (6.9%)	3 (5.2%)	19 (32.8%)	3 (5.2%)	

Table S6. Subgroups analysis concerning the awareness of the risk of complications.

		Same risk N (%)	*No opinion N (%)*	*Higher risk N (%)*	*p value*
During the acute phase	***Age***				
	< 65 y	12 (8.8%)	26 (19%)	99 (72.3%)	0.353
	≥ 65 y	5 (5.2%)	14 (14.6%)	77 (80.2%)	
	Sex				
	Male	6 (6.7%)	15 (16.9%)	68 (76.4%)	0.959
	Female	11 (7.6%)	25 (17.4%)	108 (75%)	
	Treatment intent				
	Curative	7 (8.1%)	17 (19.8%)	62 (72.1%)	0.643
	Palliative	10 (6.8%)	23 (15.6%)	114 (77.6%)	
	Type of treatment				
	Chemotherapy	4 (3.8%)	16 (15.1%)	86 (81.1%)	0.511
	Immunotherapy	6 (10.7%)	10 (17.9%)	40 (71.4%)	
	Targeted therapy	4 (11.1%)	8 (22.2%)	24 (66.7%)	
	Combination	3 (8.6%)	6 (17.1%)	26 (74.3%)	
	Cancer site				
	Breast	3 (5.6%)	12 (22.2%)	39 (72.2%)	**0.015**
	Lung	1 (1.8%)	7 (12.3%)	49 (86%)	
	Gastrointestinal	1 (2.2%)	7 (15.2%)	38 (82.6%)	
	Other	12 (15.8%)	14 (18.4%)	50 (65.8%)	
During the post-acute phase	***Age***				
	< 65 y	11 (10%)	16 (14.5%)	83 (75.5%)	0.190
	≥ 65 y	16 (19%)	12 (14.3%)	56 (66.7%)	
	Sex				
	Male	13 (17.6%)	11 (14.9%)	50 (67.6%)	0.487
	Female	14 (11.7%)	17 (14.2%)	89 (74.2%)	
	Treatment intent				
	Curative	13 (17.8%)	7 (9.6%)	53 (72.6%)	0.203
	Palliative	14 (11.6%)	21 (17.4%)	86 (71.1%)	
	Type of treatment				
	Chemotherapy	8 (9.5%)	9 (10.7%)	67 (79.8%)	**0.047**
	Immunotherapy	13 (25%)	11 (21.2%)	28 (53.8%)	
	Targeted therapy	4 (12.9%)	5 (16.1%)	22 (71%)	
	Combination	2 (7.4%)	3 (11.1%)	22 (81.5%)	
	Cancer site				
	Breast	7 (15.2%)	6 (13%)	33 (71.7%)	0.089
	Lung	4 (7.7%)	8 (15.4%)	40 (76.9%)	
	Gastrointestinal	3 (9.4%)	1 (3.1%)	28 (87.5%)	
	Other	13 (20.3%)	13 (20.3%)	38 (59.4%)	
